# Neglected Lunate Dislocation: A Case Report on Successful Management With Open Reduction and Internal Fixation

**DOI:** 10.7759/cureus.86257

**Published:** 2025-06-18

**Authors:** Mohammed Ali, Ahmed Alazzawi, Mohammed A Alwatari, Abdullah Zalzala, Mohammed Alghannami

**Affiliations:** 1 Orthopaedic Surgery, Al Farahidi University, Baghdad, IRQ; 2 Orthopaedic Surgery, Al-Wasity Teaching Hospital, Baghdad, IRQ; 3 Orthopaedic Surgery, College of Medicine, University of Baghdad, Baghdad, IRQ

**Keywords:** kirschner wire, lunate dislocation, neglected, orif, wrist arthrodesis

## Abstract

Lunate dislocations are often underdiagnosed wrist injuries that typically result from a fall onto an outstretched hand. If left untreated, they may lead to significant functional impairment.

We report the case of a 65-year-old man who presented with persistent wrist pain six months after a fall. The initial diagnosis was missed. Radiographs and computed tomography (CT) revealed a neglected volar lunate dislocation. Surgical management with open reduction and internal fixation (ORIF) using Kirschner wires was performed under regional anesthesia. The patient experienced substantial pain relief and improved wrist function postoperatively. This case underscores the importance of early diagnosis and surgical intervention while demonstrating that favorable outcomes can still be achieved in delayed presentations.

## Introduction

Lunate dislocations are relatively uncommon injuries in acute wrist trauma. Literature suggests that 7% of all carpal injuries involve the lunate, and only 3% are classified as volar lunate dislocations. Misdiagnosis occurs in approximately 25% of cases [1,2].

The typical mechanism involves a fall onto a hyperextended wrist with the forearm supinated. Mayfield et al. described a four-stage progression of carpal instability: beginning with scapholunate dissociation, followed by perilunate dislocation, midcarpal dislocation, and finally, lunate dislocation, the rarest and most severe stage [3].

Management of lunate dislocation remains controversial and largely dependent on physician preference. Options include closed reduction, ligamentous repair with open reduction and internal fixation (ORIF), proximal row carpectomy, and wrist arthrodesis in chronic cases [4,5].

## Case presentation

A 65-year-old man presented with persistent wrist pain lasting three months following a fall onto an outstretched hand. Initially misdiagnosed in the emergency department, his primary complaint was ongoing wrist pain without noticeable limitations in movement. Clinical examination revealed tenderness but preserved range of motion.

**Figure 1:**
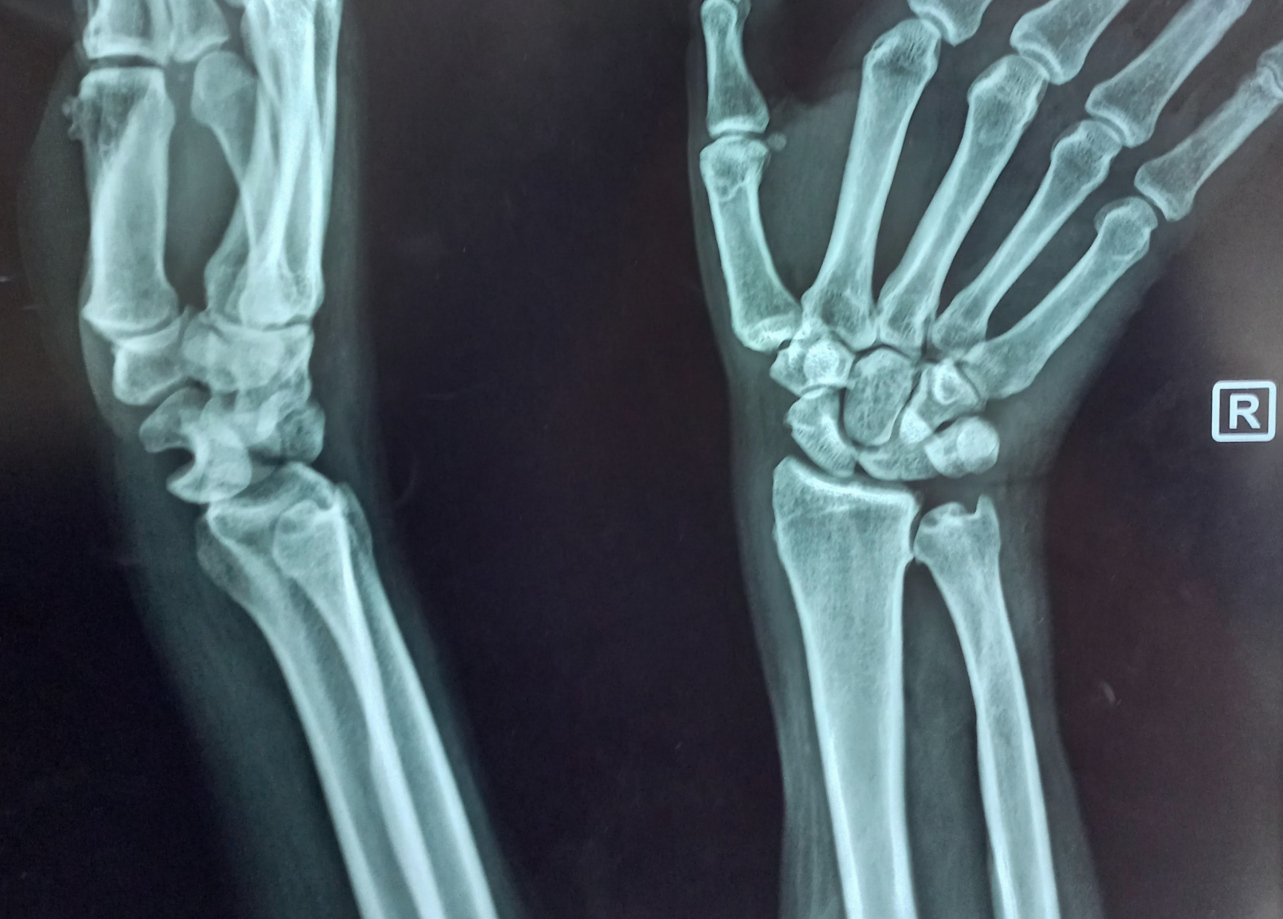
Preoperative posteroanterior radiograph showing disruption of Gilula’s arc, and lateral view demonstrating the “spilled teacup” sign.

The posteroanterior radiograph demonstrated a break in Gilula's arc and the lateral radiograph demonstrated the classic “spilled teacup” sign, indicative of a neglected lunate dislocation (Figure 1).

**Figure 2:**
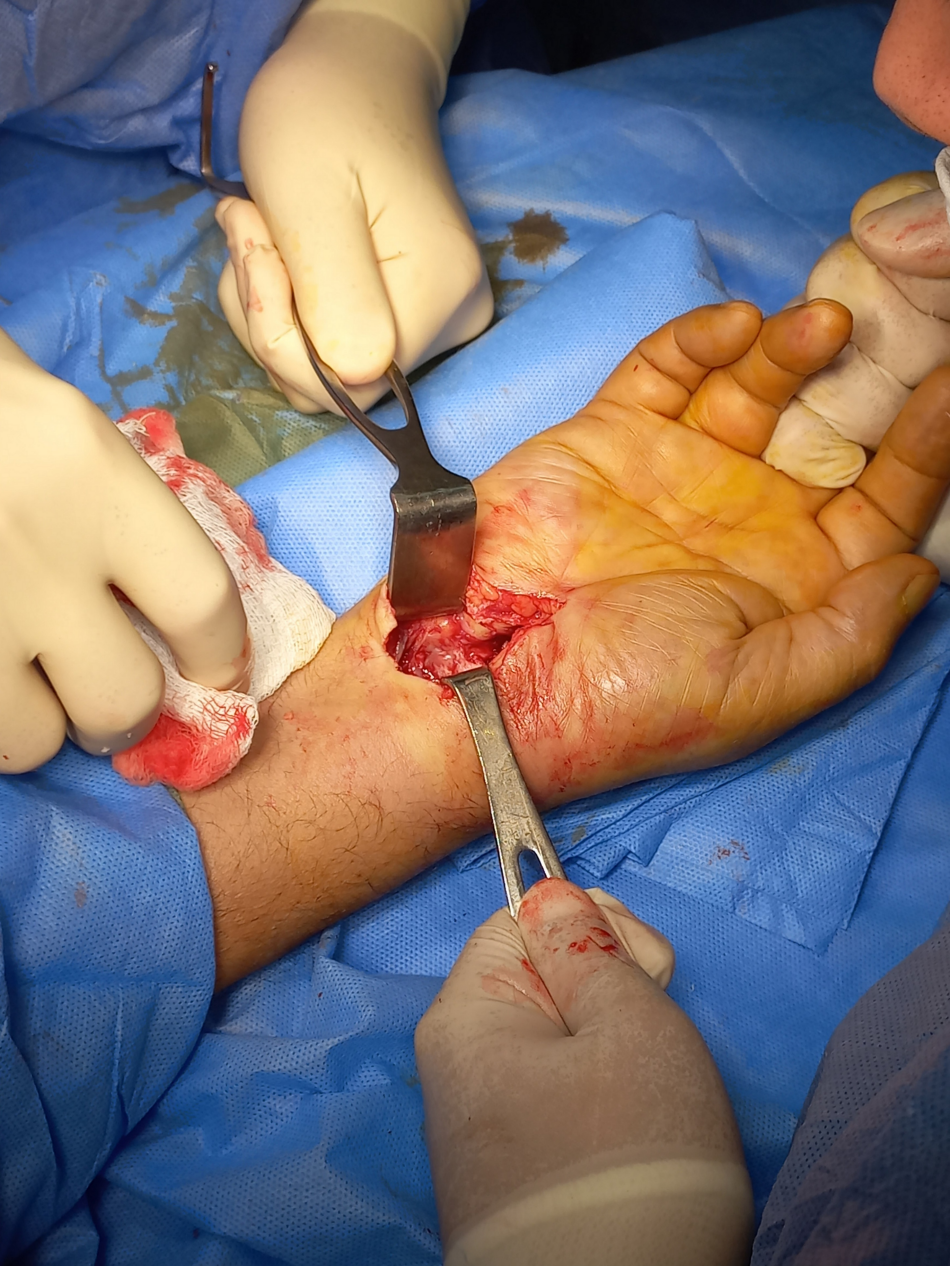
Intraoperative image during open reduction.

The patient underwent ORIF under regional anesthesia due to comorbidities (Figure 2).

**Figure 3:**
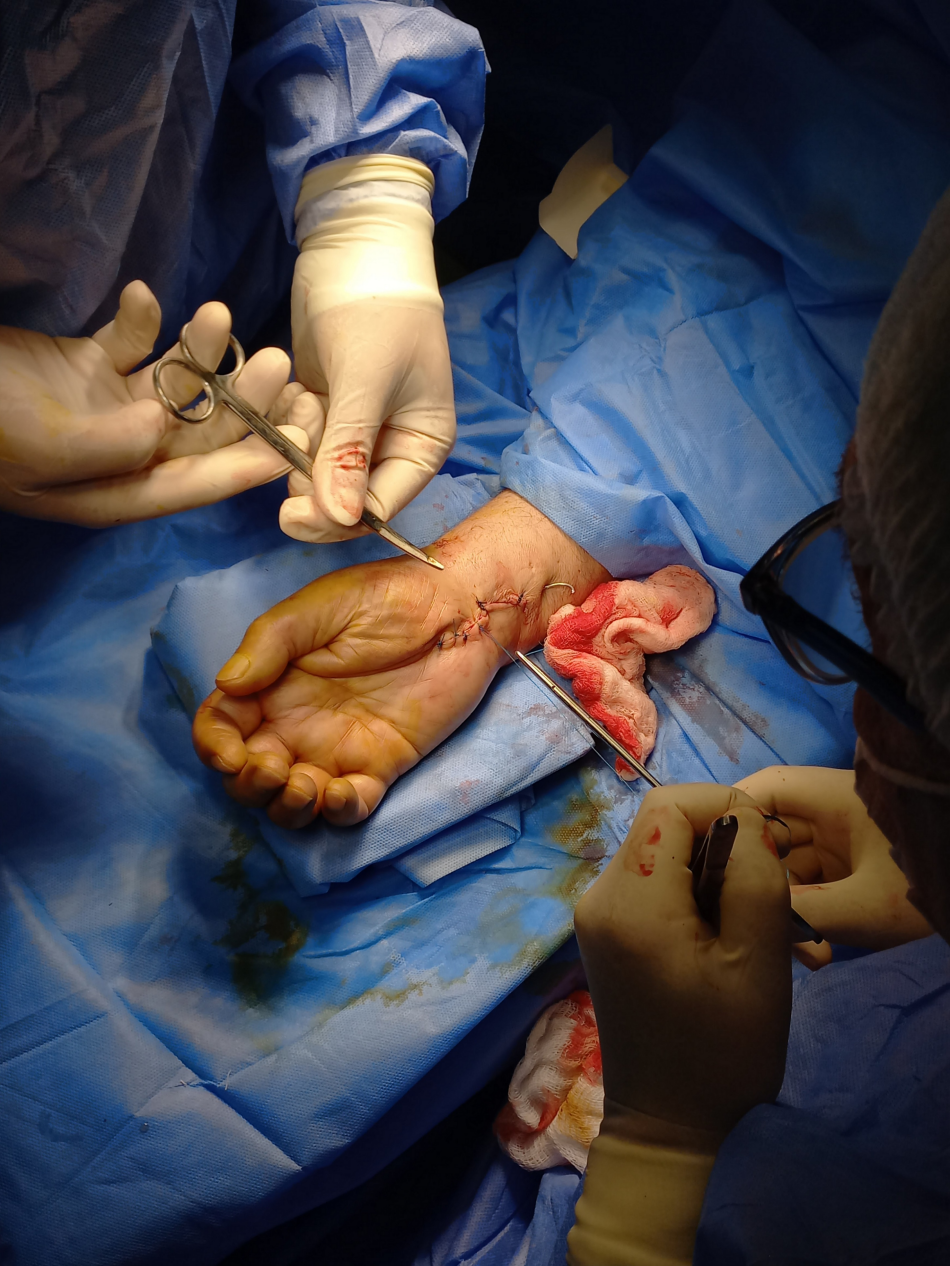
Postoperative image showing Kirschner wire fixation through a minimal incision.

The lunate was anatomically repositioned and stabilized using Kirschner wires with minimal incision (Figure 3).

The postoperative course was uneventful. The patient reported significant pain relief and improved wrist function. At 12-month follow-up, the patient had achieved satisfactory functional recovery, with an overall recovery time of approximately eight months. These outcomes are consistent with previous reports [6,7].

## Discussion

Lunate dislocations are frequently misdiagnosed, with up to 25% of cases initially missed [1,2]. The most critical factor in the misdiagnosis is the lack of clinical experience; other contributing factors include inadequate radiographs and suboptimal imaging techniques [8].

Early diagnosis and intervention are essential, as neglected lunate dislocations can lead to complications such as median nerve compression, carpal instability, avascular necrosis, complex regional pain syndrome, post-traumatic arthritis, and poor functional outcomes [9,10].

Ligament restoration plays a crucial role in re-establishing wrist stability and preventing long-term instability [9]. In the present case, fortunately, no ligamentous injuries were identified. ORIF can be performed using either a volar, dorsal, or combined (dual) approach [10]. In this case, a volar approach was utilized.

Despite delayed presentation, ORIF with Kirschner wires can yield favorable results. In our case, the procedure performed six months post-injury led to significant improvement, reaffirming the importance of anatomical reduction and stabilization in achieving functional recovery. Excellent outcomes following delayed presentation have also been documented in several other studies [6,7].

Alternative treatment modalities for chronic lunate dislocation include, firstly, staged external fixation, which facilitates realignment and soft tissue stretching prior to definitive surgery. Another option is proximal row carpectomy, considered when reduction is unfeasible or in the presence of arthritis, provided joint surfaces are preserved. A further option is wrist arthrodesis, which offers pain relief and joint stability but results in loss of mobility, making it less acceptable to some patients [6,7,10].

## Conclusions

Volar lunate dislocations are rare and frequently misdiagnosed injuries that require a high index of suspicion and proper imaging for timely diagnosis. Even in delayed cases, surgical management with ORIF can achieve favorable functional outcomes. This case highlights the importance of individualized surgical planning and reinforces the viability of ORIF in chronic lunate dislocations. Volar lunate dislocations are rare and frequently misdiagnosed injuries that require a high index of suspicion and proper imaging for timely diagnosis. Even in delayed cases, surgical management with open reduction and internal fixation can achieve favorable functional outcomes. This case highlights the importance of individualized surgical planning and reinforces the viability of ORIF in chronic lunate dislocations.
